# GPX8^+^ cancer-associated fibroblast, as a cancer-promoting factor in lung adenocarcinoma, is related to the immunosuppressive microenvironment

**DOI:** 10.1186/s12920-024-01832-8

**Published:** 2024-03-21

**Authors:** Ying Bai, Tao Han, Yunjia Dong, Chao Liang, Lu Gao, Yafeng Liu, Jiawei Zhou, Jianqiang Guo, Deyong Ge, Jing Wu, Dong Hu

**Affiliations:** 1 Key Laboratory of Industrial Dust Prevention and Control & Occupational Safety and Health of the Ministry of Education, Anhui University of Science and Technology, Huainan, Anhui China; 2 School of Medicine, Anhui University of Science and Technology, Huainan, Anhui China; 3 Anhui Occupational Health and Safety Engineering Laboratory, Huainan, Anhui China; 4 Key Laboratory of Industrial Dust Deep Reduction and Occupational Health and Safety of Anhui Higher Education Institute, Huainan, Anhui China

**Keywords:** Lung adenocarcinoma, GPX8, Cancer-associated fibroblasts, Prognosis, Immunosuppressive microenvironment

## Abstract

**Background:**

Cancer-associated fibroblasts (CAFs) play a crucial role in the tumor microenvironment of lung adenocarcinoma (LUAD) and are often associated with poorer clinical outcomes. This study aimed to screen for CAF-specific genes that could serve as promising therapeutic targets for LUAD.

**Methods:**

We established a single-cell transcriptional profile of LUAD, focusing on genetic changes in fibroblasts. Next, we identified key genes associated with fibroblasts through weighted gene co-expression network analysis (WGCNA) and univariate Cox analysis. Then, we evaluated the relationship between glutathione peroxidase 8 (GPX8) and clinical features in multiple independent LUAD cohorts. Furthermore, we analyzed immune infiltration to shed light on the relationship between GPX8 immune microenvironment remodeling. For clinical treatment, we used the tumor immune dysfunction and exclusion (TIDE) algorithm to assess the immunotherapy prediction efficiency of GPX8. After that, we screened potential therapeutic drugs for LUAD by the connectivity map (cMAP). Finally, we conducted a cell trajectory analysis of GPX8^+^ CAFs to show their unique function.

**Results:**

Fibroblasts were found to be enriched in tumor tissues. Then we identified GPX8 as a key gene associated with CAFs through comprehensive bioinformatics analysis. Further analysis across multiple LUAD cohorts demonstrated the relationship between GPX8 and poor prognosis. Additionally, we found that GPX8 played a role in inducing the formation of an immunosuppressive microenvironment. The TIDE method indicated that patients with low GPX8 expression were more likely to be responsive to immunotherapy. Using the cMAP, we identified beta-CCP as a potential drug-related to GPX8. Finally, cell trajectory analysis provided insights into the dynamic process of GPX8^+^ CAFs formation.

**Conclusions:**

This study elucidates the association between GPX8^+^ CAFs and poor prognosis, as well as the induction of immunosuppressive formation in LUAD. These findings suggest that targeting GPX8^+^ CAFs could potentially serve as a therapeutic strategy for the treatment of LUAD.

**Supplementary Information:**

The online version contains supplementary material available at 10.1186/s12920-024-01832-8.

## Introduction

Lung cancer is one of the leading causes of death in cancer patients in the world today [1]. Lung adenocarcinoma, the most common histologic subtype of cancer, is responsible for approximately 40% of all lung cancer diagnoses [2]. Advances in early diagnostic techniques and more sophisticated, systematic surgical resection and chemotherapy have improved survival times and quality of life for patients with LUAD [3, 4]. However, due to the malignant nature of lung adenocarcinoma, which is prone to metastasis and drug resistance, the 5-year survival rate is still only around 20% [5–7]. Therefore, it is essential to investigate the mechanisms that drive the progression of LUAD and to identify viable therapeutic targets.

The remodeling of the tumor microenvironment is widely acknowledged as a critical factor that influences the effectiveness of clinical anticancer therapy [8]. As a major component of the tumor microenvironment, CAFs play a crucial role in promoting the progression of malignant tumors. This role is diverse and involves various functions such as regulating tumor cell growth, migration, and invasion [9, 10]. CAFs have been shown to not only modulate growth, metastasis, and angiogenesis by directly regulating cancer cells [11, 12], but also can indirectly induce the formation of a microenvironment conducive to tumor invasion by activating and recruiting non-tumor components in the TME [13]. For example, CAFs can induce the recruitment of myeloid cells, which in turn induces immunosuppression of T cells and angiogenesis [14–16]. In co-culture, CAF was found to inhibit NK cell activation by interfering with IL-2-mediated upregulation of the triggering receptors NKp44, NKp30, and DNAM-1 [17]. Additionally, CAFs secrete a variety of molecules in the TME that assist in LUAD progression and metastasis, including matrix metalloproteinases (MMPs family), cytokines, chemokines, and growth factors among others [13]. CAF has begun to emerge as a key target for novel cancer therapies in the clinical setting. Currently, more and more drugs targeting CAF activation-related pathways have begun to enter the clinical field, such as TGF-β inhibitors, FAK inhibitors, Hedgehog inhibitors, and fibroblast growth factor receptor (FGFR) inhibitors [17]. Therapeutic strategies targeting CAF have been shown to be effective in the treatment of non-small cell lung cancer (NSCLC) [18]. Therefore, exploring targets associated with CAFs represents a promising therapeutic strategy for LUAD treatment.

Glutathione peroxidase 8 (GPX8) is a transmembrane protein localized in the ER and the last recognized member of the glutathione peroxidase (GPX) protein family, which has the primary function of limiting the cellular accumulation of reactive oxygen species (ROS) [19, 20]. GPX8 can reduce oxidized PDI and prevent endoplasmic reticulum oxidoreductase 1 alpha (ERO1α)-derived H_2_O_2_ leakage by regulating ERO1α [21, 22]. Recent studies have shown that GPX8 can maintain the invasive mesenchymal-like phenotype of breast cancer cells through the IL-6/STAT3 axis [23]. GPX8 has been recognized as a prognostic marker for cancers such as gastric cancer and primary glioma [24, 25]. In clear cell renal cell carcinoma, GPX8 silencing inhibits tumorigenesis by regulating nicotinamide N-methyltransferase (NNMT) [26]. These studies demonstrated the importance of aberrant expression of GPX8 in carcinogenesis, which may serve as a potential target for cancer therapy. However, the oncogenic role of GPX8 in LUAD still needs to be further explored.

Our current study, based on single-cell sequencing data and comprehensive analysis of multiple independent bulk-RNA cohorts, has identified that GPX8 is expressed aberrantly in LUAD and is primarily found in CAFs, where it plays a key role in tumor progression. We found that the activation of GPX8 in the tumor microenvironment is associated with poorer survival outcomes and the development of an immunosuppressive environment. Functionally, GPX8^+^ CAFs demonstrated enhanced cell adhesion, migration, and immunomodulatory capabilities compared to GPX8^−^ CAFs. Together, these findings suggest that the interplay between GPX8 and CAFs significantly influences LUAD tumor progression, and GPX8^+^ CAFs could represent a novel therapeutic target for future CAF-directed treatments.

## Materials and methods

### Data downloading and preprocessing

Bulk-RNA sequencing data: mRNA expression profiles and clinical information of The Cancer Genome Atlas Program (TCGA), LUAD patient data, including 576 samples, 517 tumor samples, and 59 normal samples, were obtained from the UCSC website (https://xenabrowser.net/datapages/). Transcriptome data and clinical information for GSE31210, GSE72094, GSE30219, GSE50081, and GSE19188 were obtained from the Gene Expression Omnibus (GEO) database (https://www.ncbi.nlm.nih.gov/geo/).

Download and integration of single-cell RNA sequencing data: scRNA-seq data for two LUADs (GSE123902 and GSE153935) were obtained from the GEO database, respectively. 17 samples were acquired from GSE123902, 4 normal and 13 tumor samples. 18 samples were obtained from GSE153935, which were 6 normal and 12 tumor samples. The screening criteria for the cells were as follows: a). each sample should contain no less than 300 and no more than 5000 cells; b). each cell should express more than 200 genes; c). each gene should be expressed in at least 3 cells; and d). the content of mitochondrial RNA in each cell should be less than 30% [27]. After filtering the qualified cells are screened by these criteria, we used the “NormalizeData” function in the Seurat package to normalize the expression values. Subsequently, the “FindVariableFeatures” function was used to identify 2000 highly variable genes, which were then centered using the “ScaleData” function [27]. Finally, we used the “harmony” R package [28] for data integration. The “harmony” R package enables fast, sensitive and accurate integration of cells from different donors, tissues and technology platforms.

### Cellular annotation, differential gene, and functional enrichment analysis

In this study, we utilized the “SingleR” package [29] to automatically annotate the samples by cell type and combined it with manual annotation. The SingleR package calculates the correlation between the gene expression of a single cell sample and the gene expression of a cell type from a reference database. By iteratively eliminating the weakest correlation for each cell type, the corresponding cell type can be identified [29]. Furthermore, considering that the differences in the study population may introduce some bias to the results of the automatic annotation, we then further manually annotated the automatically annotated cell populations. Subsequently, we used the “FindAllMarkers” function in the “Seurat” package to identify differentially expressed genes (DEGs) for further analysis by applying a threshold value of |log2FoldChange| > 0.8 and the adjusting *P* value < 0.05, and then used the “ClusterGVis” R package (https://github.com/junjunlab/ClusterGVis) for visualization. “ClusterGVis” R package was used to visualize the heatmap of differential genes. The “clusterProfiler” R package was used for Gene Ontology (GO) enrichment analysis and Kyoto Encyclopedia of Genes and Genomes (KEGG) enrichment analysis.

### Estimation of CAFs score in LUAD

We obtained marker genes for CAFs from a previous study [30] and evaluated these marker genes using the single sample gene set enrichment analysis (ssGSEA) to assess the CAFs in LUAD. In addition, the EPIC algorithm [31] was also utilized in order to assess the amount of CAFs in patients. Subsequently, K-M curve survival analysis was used for the prognostic value of CAFs assessed by different algorithms.

### Identification of CAFs-related key genes in LUAD

We used weighted gene co-expression network analysis (WGCNA) to identify key gene expression networks associated with the CAFs in 3 independent cohorts: TCGA, GSE31210 and GSE72094. We screened the top 75% genes by of the median absolute deviation (MAD) for network construction (The MAD method can screen for genes with high variability that are representative of the sample’s attributes), After that, we adopted the criteria of the previous study [32] using the standard method cutreeDynamic function to identify co-expressed gene modules with a minimum module size of 30 and a merge height cut of 0.25. The association between modules) and CAFs scores was assessed by correlation analysis, *p*-values < 0.05 were considered significant, and modules highly correlated with CAFs scores were selected for further analysis. Subsequently, we intersected CAFs-related genes obtained from the 3 LUAD bulk-RNA cohorts and the single cell data (GSE123902) by overlapping genes. Then, we performed a univariate Cox analysis of these genes in each of the 3 cohorts to obtain prognostically relevant key genes. Next, the Tumor Immune Single-cell Hub 2 (TISCH2, http://tisch.comp-genomics.org/home/) and the scRNASeqDB database (https://bioinfo.uth.edu/scrnaseqdb/) were used to validate the expression profiles of key genes in CAFs. The UALCAN database (https://ualcan.path.uab.edu/) was used to assess the protein expression changes of key genes, and the cBioportal database (https://www.cbioportal.org/) was used to analyze the mutation frequency.

### Analysis of copy number variation

GSCALiter is a comprehensive pan-cancer genomic web server (http://bioinfo.life.hust.edu.cn/web/GSCALite/). We used GSCALite public server to analyze the copy number variation (CNV) of GPXs family (GPX1-GPX8) at the pan-cancer level.

### Determination of TME cell infiltration levels and immune-related functional enrichment scores

We evaluated the ESTIMATE, immune and stromal scores of tumor samples in TCGA using the “ESTIMATE” method. Tumor purity scores of TCGA-LUAD patients, 29 TME signature genes, as well as tumor microenvironmental properties and immune subtypes of LUAD patients were obtained from previous studies [30, 33]. With the CIBERSORT algorithm, we obtained the infiltration levels of each of the 22 immune cell types. In addition, we evaluated the TME-related scores summarized by Zeng et al. [34].

### Enrichment analysis for hallmark gene sets

Hallmark gene sets were obtained from the Molecular Signatures Database (MSigDB, https://www.gsea-msigdb.org/gsea/msigdb), which were assessed and quantified using ssGSEA. In addition, the “clusterProfiler” R package was used for GSEA analysis and “GseaVis” R package (https://github.com/junjunlab/GseaVis/wiki) was used for visualization.

### Immune checkpoints and tumor immune dysfunction and exclusion (TIDE) analysis

Correlation analysis was used to calculate the correlation between GPX8 and common immune checkpoints, which was visualized using the “corrplot” R package. The TIDE algorithm is mainly based on two mechanisms of tumor immune evasion: (1) the induction of T-cell dysfunction in tumors with high cytotoxic T-lymphocyte (CTL) infiltration; (2) the prevention of T-cell infiltration in tumors with low CTL levels. After uploading scaled transcriptome expression profiles, TIDE scores and immunotherapy responses of patients in the LUAD cohorts were calculated by TIDE website (http://tide.dfci.harvard.edu).

### Tissue immunofluorescence analysis

After paraffin embedding, 5 μm sections are deparaffinized, rehydrated, and antigen restored, and the primary antibody added prior to BSA blocking is treated with EDTA Antigen Repair Buffer. Sections were incubated overnight at 4 °C in a wet room, washed, and then the secondary antibody was dropped into the overlying tissue and incubated at room temperature. Cell nuclei were restained with DAPI, blocked and photographed. Anti - α-SMA Rabbit pAb (GB111364-100) was purchased from Servicebio; Anti – GPX8 Rabbit pAb (GTX125992) was purchased from GeneTex.

### Screening for small molecule drug candidates for GPX8

We performed differential gene analysis on patients from the TCGA, GSE72094, and GSE31210 datasets separately. Firstly, the patients were divided into high and low groups based on the median value of GPX8 expression. Next, to identify differentially expressed genes (DEGs), we utilized the ‘limma’ R package [35] to analyze the DEGs in different GPX8 groups among the 3 cohorts. The DEGs were determined using |log2FoldChange| > 1 and adj. *p* value < 0.05 as the criteria, and then we performed an intersection of the DEGs. To identify potential GPX8 related therapeutic agents, we imported the final intersected DEGs into the connectivity map (cMAP) database (https://clue.io/).

### Cell trajectory analysis (pseudotime analysis)

We used the “monocle2” package to perform pseudotime analysis of fibroblast cell subpopulations [36]. To explore the differentiation trajectories and related genes between different states of fibroblast cell subpopulations, the “plot_cell_trajectory” function was used to sort cells according to their pseudotimes. The “BEAM” function was used to identify genes responsible for cell branching and differentiation. The results were visualized using the “plot_genes_branched_heatmap” function.

### Statistical analysis

Wilcoxon rank sum test was utilized to assess statistical significance between different groups. Kaplan-Meier survival curves were used to analyze the survival of different LUAD patients. Pearson or spearman correlation analysis was used for correlation analysis. Statistical significance was described as follows: ns (not significant), * *p* < 0.05, ** *p* ≤ 0.01, *** *p* ≤ 0.001.

## Results

### Cancer-associated fibroblasts are associated with poor prognosis in lung adenocarcinoma

**Fig. 1:**
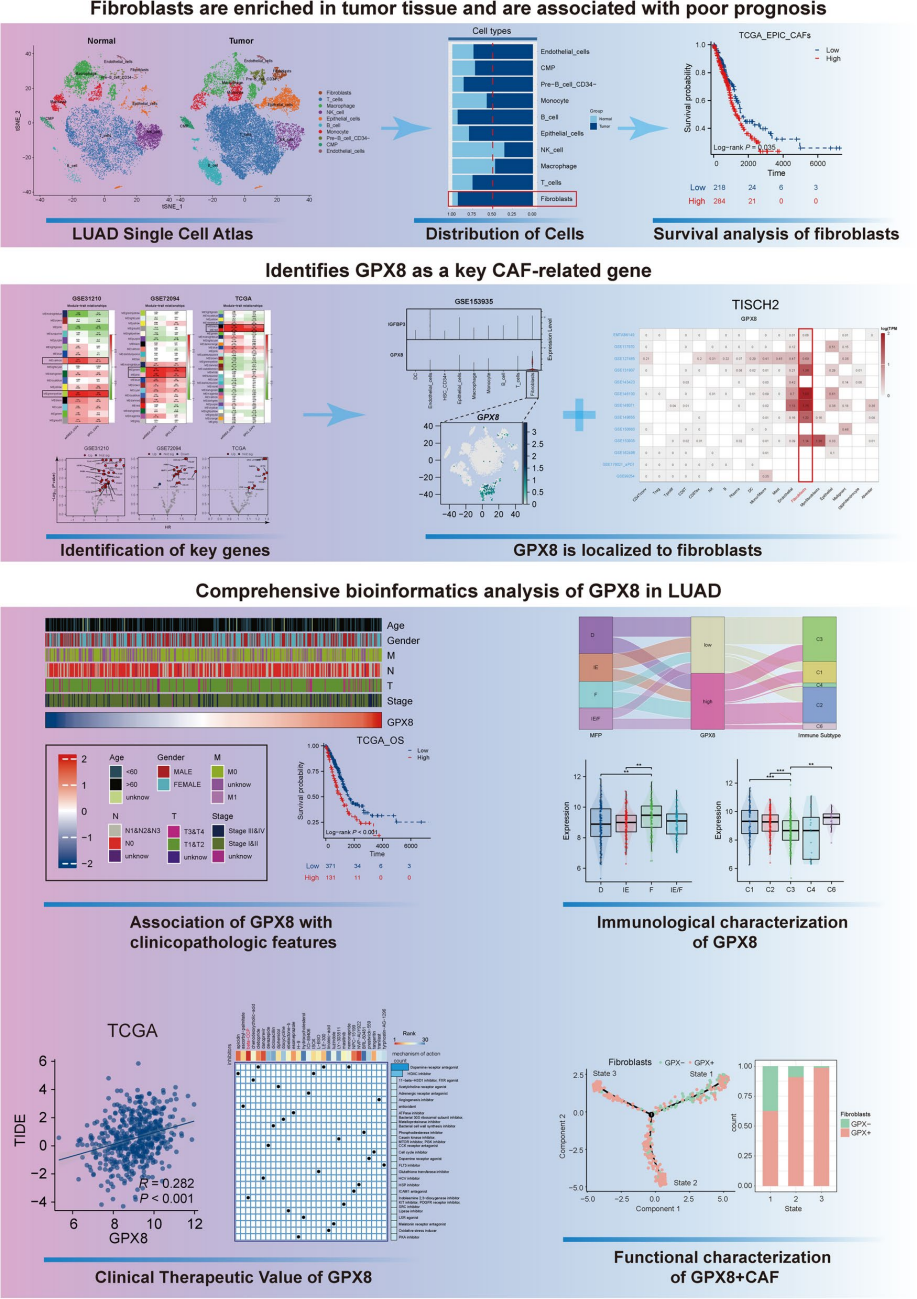
The overview of study design to evaluate the biological role of GPX8^+^ CAFs in lung adenocarcinoma

**Fig. 2:**
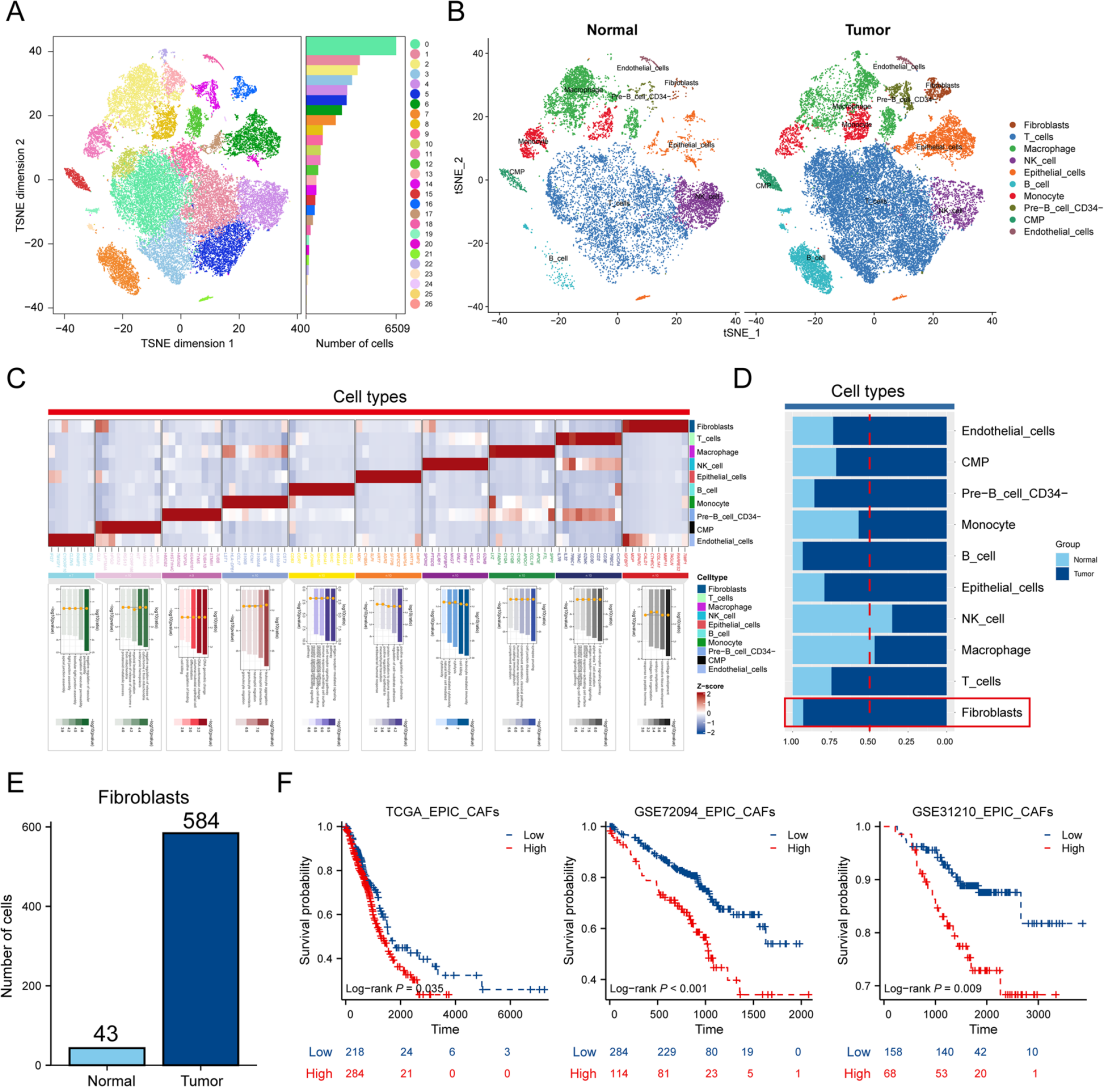
Overview of single-cell data for normal and tumor samples. **A** t-SNE clustering plots of 17 samples. **B** t-SNE clustering plot of normal and tumor tissues. **C** Heatmap showing marker genes and biological pathways involved in 10 cell types. **D** Stacked diagram of cell distribution in normal and tumor tissues. **E** Bar plot showing the number of fibroblasts in normal and tumor tissues. **F** Quantification of CAFs abundance in LUAD using the EPIC algorithm, and analysis of the prognostic value of CAFs using the K-M curve analysis

Figure 1 presents the workflow diagram of the study conducted. In the initial step, we analyzed 17 samples from the GSE123902 dataset, consisting of 4 normal tissues and 13 tumor tissues. Subsequently, we screened each sample to eliminate low-quality data, resulting in the retention of 38,124 cells for further analysis. Clustering visualization using the t-SNE method revealed the distinction of cells into 27 clusters (Fig. 2A). To refine the annotation, we employed the “SingleR” and manual annotation, classifying the cells into 10 distinct types (Fig. 2B). Among them, tumor tissues exhibited a higher abundance of adaptive response immune cells (T cells, B cells) relative to immune cell types involved in the innate immune response (macrophages, NK cells). This result may attribute to the antigenic heterogeneity in tumor cells (Fig. 2B). To gain further insight into the genetic aspects, we constructed a heatmap showcasing the Top10 differential genes and the Top5 enriched biological pathways for each cell type (Fig. 2C). And it’s worth noting that we found that more fibroblasts were recruited at the cancer tissue (Fig. 2D, E). Evaluating the extent of cancer-associated fibroblasts (CAFs) infiltration in the bulk-RNA cohorts, the prognostic analysis revealed that a higher level of CAFs correlated with decreased survival time in patients (Fig. 2F) (Supplementary Fig. 1A). These findings suggest that CAFs are enriched in tumor tissues and contribute to tumor progression.

### Identification of GPX8 as a key prognostic gene associated with CAFs

**Fig. 3:**
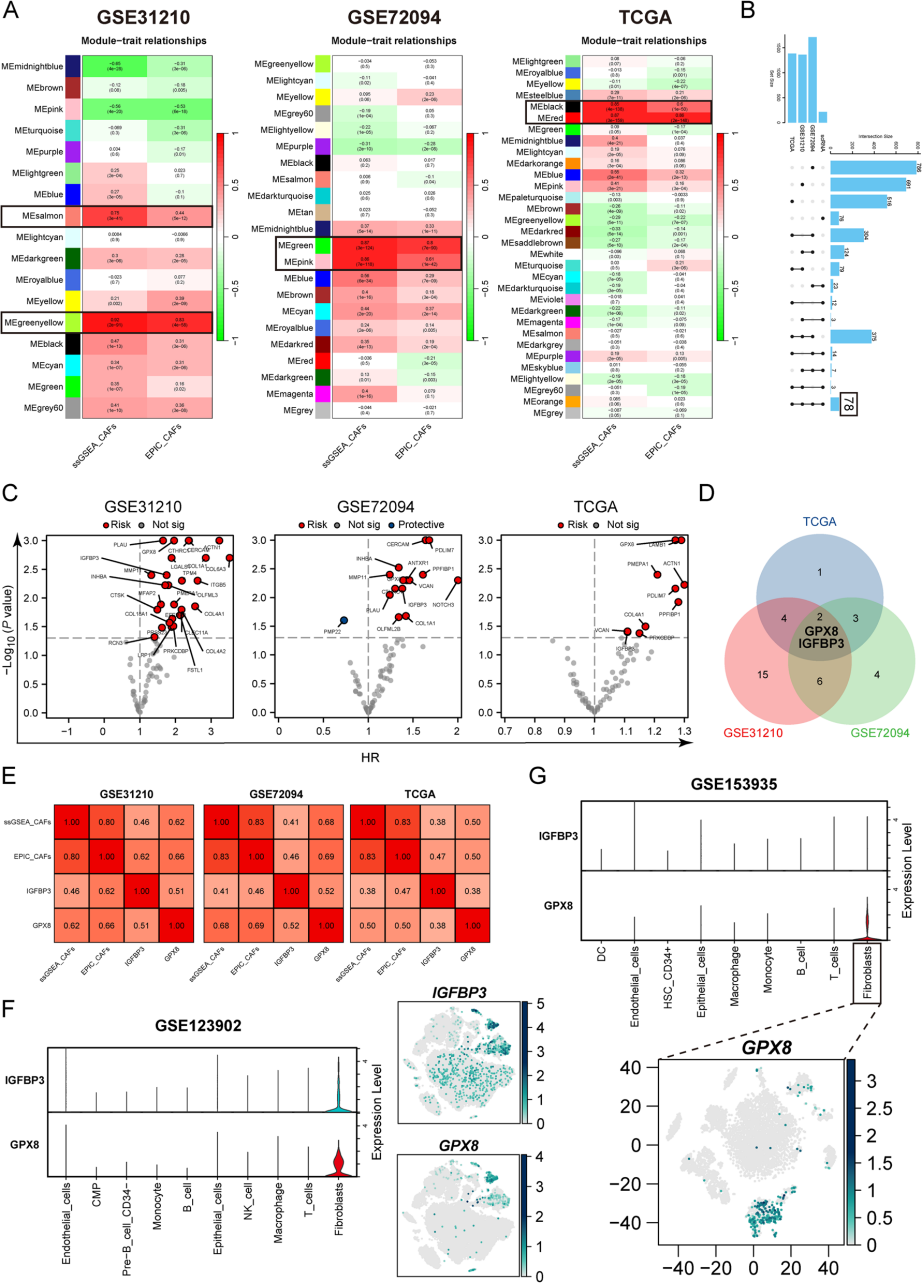
GPX8 promotes cancer progression. **A** Correlation heatmap showing the correlation between EPIC_CAFs and ssGSEA_CAFs with gene modules in the 3 LUAD cohorts. **B** Upset plot showing the overlap of fibroblast-associated genes from the 3 LUAD cohorts and the single-cell data (GSE123902). **C** Volcano plots were used to visualize the prognostic value of genes. **D** Venn plots overlapping CAFs-related prognostic genes of the 3 cohorts. **E** Heatmap of the correlation of GPX8 and IGFBP3 with CAFs. **F** Violin plots and scatter plots of GPX8 and IGFBP3 expression in the GSE123902 dataset. **G** Violin plots and scatter plots of GPX8 and IGFBP3 expression in the GSE153935 dataset

Afterward, we identified key prognostic genes associated with cancer-associated fibroblasts (CAFs) using bulk-RNA data. We performing WGCNA analysis for each of the three LUAD cohorts to identify gene co-expression network modules (Supplementary Fig. 2A, B). We then estimated the correlation between each module and the scores indicating CAF infiltration (ssGSEA_CAFs and EPIC_CAFs. Modules with correlations higher than 0.4 for both scores were selected, indicating a strong relationship between the module genes and CAF infiltration. Subsequently, we focused on the genes within these modules (Fig. 3A). The Upset plots demonstrated the intersection of module genes across the 3 cohorts and the single cell data (GSE123902), revealing 78 shared CAFs-associated genes (Fig. 3B). To identify prognostic genes among these, we performed univariate Cox analysis in the 3 bulk-RNA cohorts (Fig. 3C). We then overlapped the prognostic-related genes from 3 cohorts and screened for GPX8 and IGFBP3 (Fig. 3D). Correlation analysis showed that the two CAF scores had a strong correlation between GPX8, IGFBP3 and CAFs, with GPX8 showing a relatively higher correlation score compared to IGFBP3 (Fig. 3E). To further examine the expression localization, we investigated GPX8 and IGFBP3 expression on fibroblasts using single-cell sequencing data. Analysis of the GSE123902 and GSE153935 datasets demonstrated that GPX8 exhibited relatively specific and higher expression on fibroblasts compared to IGFBP3 (Supplementary Fig. 2C) (Fig. 3F, G). The specific localization of GPX8 on fibroblasts was also confirmed using the TISCH and scRNAseqDB databases (Supplementary Fig. 2D, E). Additionally, we assessed the localization of α-SMA (a biomarker for cancer-associated fibroblasts) and GPX8 through immunofluorescence in LUAD specimens. The results showed co-localization of α-SMA and GPX8 (Supplementary Fig. 3A). Altogether, the consistent findings support that GPX8 is predominantly expressed in CAFs to fulfill its biological role.

### GPX8 is associated with clinical malignant pathologic features

**Fig. 4:**
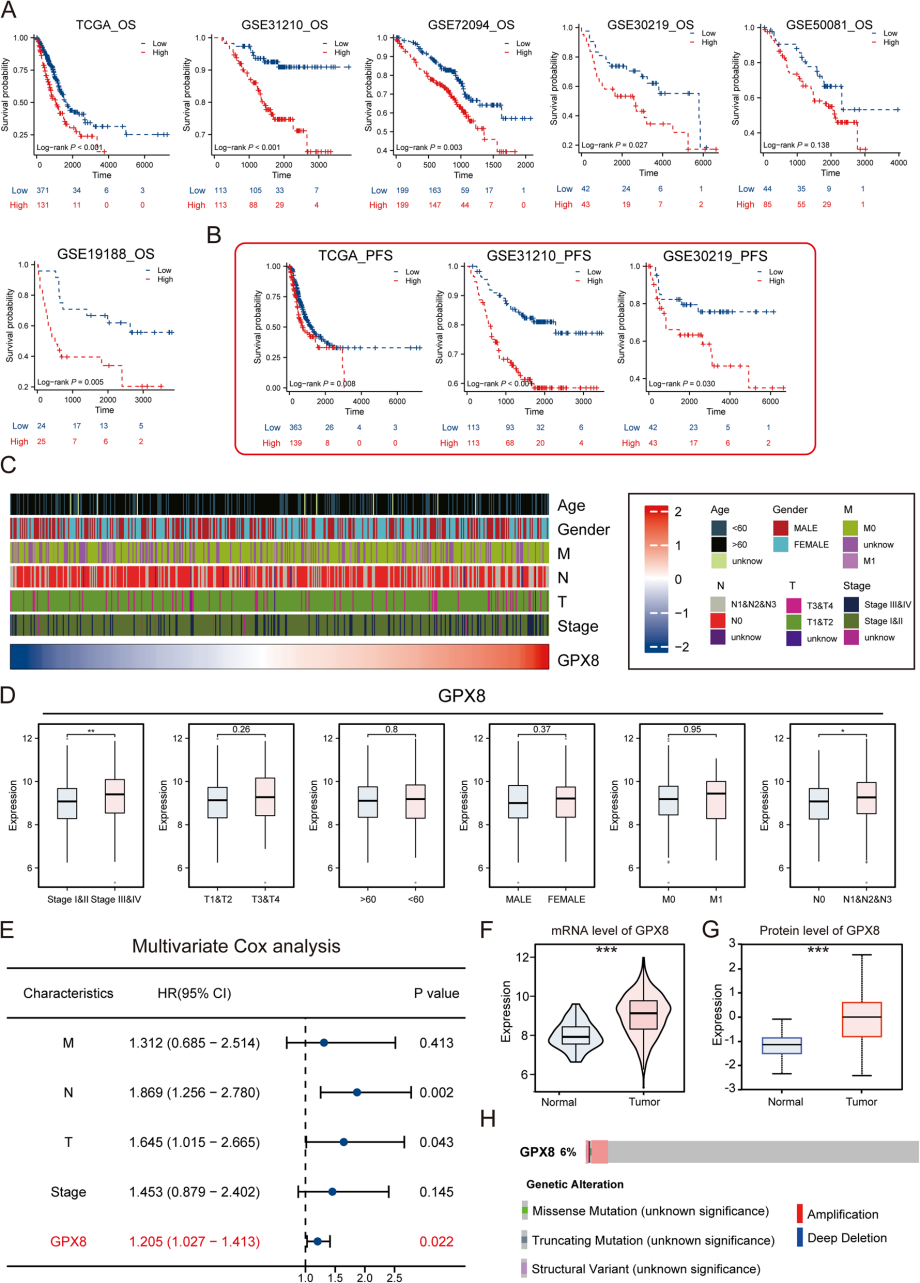
GPX8 is associated with adverse clinical outcomes. K-M curves were analyzed for OS (**A**) and PFS (**B**) of GPX8 in 6 independent cohorts. **C** Heatmap of the distribution of GPX8 with clinicopathologic features. **D** Comparison plot of GPX8 with different clinical features. **E** Forest plot of multivariate cox analysis of GPX8 in combination with other clinicopathologic features. **F** Differential expression of GPX8 at mRNA level in normal and tumor tissues in the TCGA cohort. **G** Differences in protein expression levels of GPX8 in normal and tumor tissues were analyzed using the ULCAN database. **H** Mutation profile of GPX8 was analyzed using cBioportal database (***p < 0.001; **p < 0.01; *p < 0.05; ns. no significance)

We further validated the prognostic role of GPX8 in 6 independent LUAD cohorts. As shown in (Fig. 4A, B), GPX8 exhibited strong prognostic predictive ability across different cohorts. Furthermore, the analysis incorporating clinicopathologic features indicated that high GPX8 expression correlated with more advanced clinicopathologic features (Fig. 4C, D). Multivariate Cox analysis confirmed GPX8 as an independent predictor of overall survival (OS) (HR = 1.205, *p* = 0.022) in LUAD patients (Fig. 4E). GPX8 expression at both mRNA and protein levels revealed significantly higher expression in tumor tissues compared to the normal group (Fig. 4F, G). Analysis of the cBioportal database indicated a mutation rate of 6% for GPX8 in LUAD (Fig. 4H). Additionally, a correlation analysis of the GPXs family revealed a significant expression correlation between GPX8 and GPX7 across all 3 cohorts (Supplementary Fig. 4A-C). This may be related to the functions of both enzymes, GPX7 and GPX8, which are located in the endoplasmic reticulum and together participate in the oxidative folding of endoplasmic reticulum proteins [37].

Copy number variation (CNV) is a class of structural genomic variation, which is generally considered to be one of the major factors influencing tumorigenesis and progression [38]. CNV is mainly classified as copy number heterozygous amplifications and copy number heterozygous deletions, which can induce aberrant expression of oncogenes, DNA repair genes, and other genes to influence tumor formation [39–42]. We analyzed the variation of CNV frequency among GPXs family in pan-cancer. We found that the GPXs family had universal CNV in different cancers and there was significant heterogeneity among different cancers. Among them, GPX8 showed mainly copy number heterozygous amplification in cancers such as ACC, LIHC and DLBC; while in cancers such as LUSC and ESCA, it mainly showed significant copy number heterozygous deletion. In LUAD, the GPX8 also had frequent CNV. Taken together, these results suggest that genomic instability is largely involved in the occurrence of functional abnormalities of the GPXs family in cancer. (Supplementary Fig. 5A, B).

### GPX8 is associated with the formation of immunosuppressive microenvironment

**Fig. 5:**
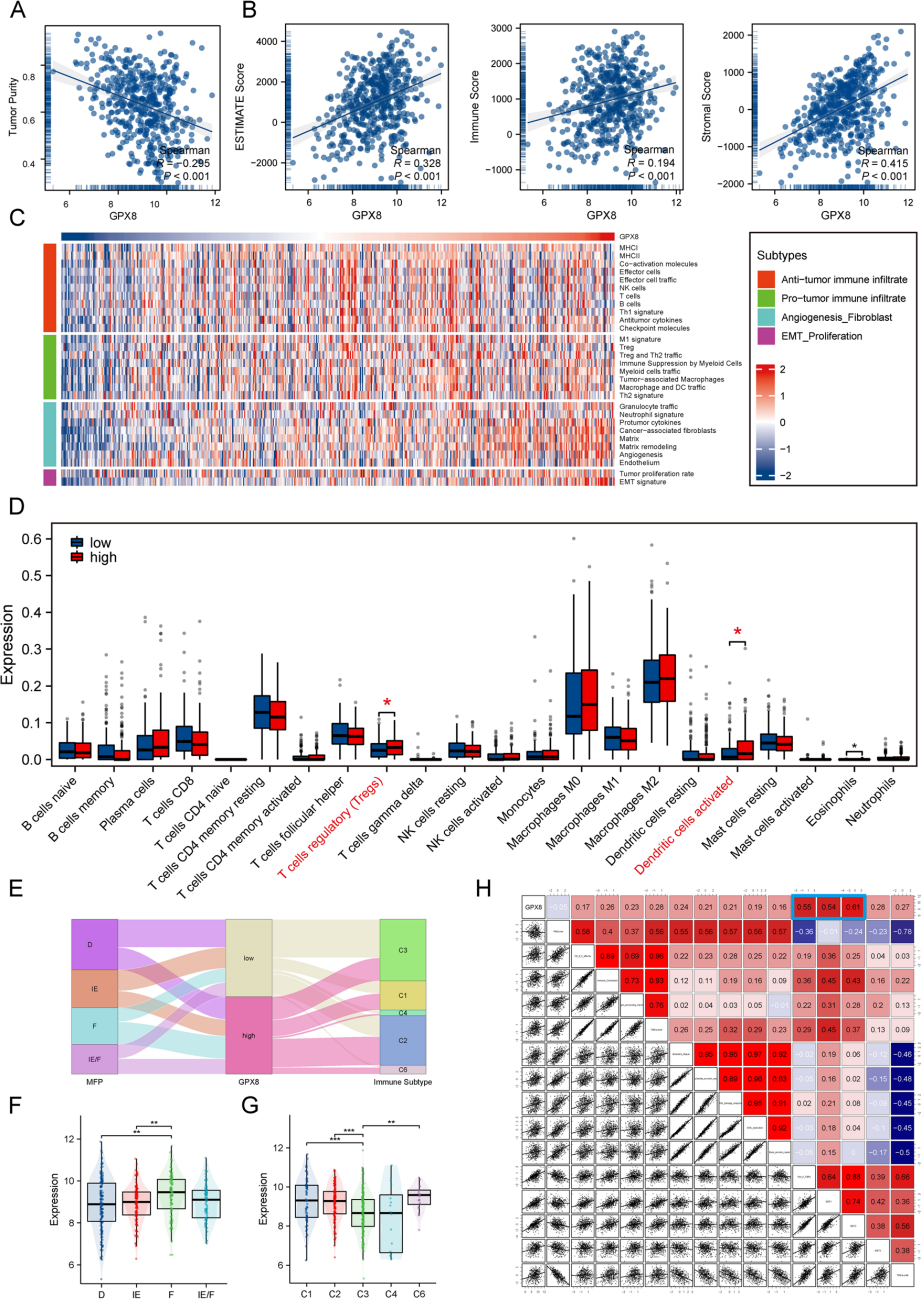
Role of GPX8 in the tumor microenvironment. **A** Correlation scatter plot of GPX8 with tumor purity. **B** Correlation scatter plot of GPX8 with ESTIMATE score, immune score, and stromal score. **C** Heatmap of the distribution of 29 TME-associated signatures with GPX8 expression values. **D** Relationship between GPX8 expression and the proportion of 22 immune cells infiltrated. **E** Sankey diagram of TME attributes and immune subtype attributes of patients in different GPX8 groups. Distribution of GPX8 expression in TME subtypes defined by Bagaev et al. (**F**) and immune subtypes defined by Thorsson et al. (**G**). **H** Heatmap of different TME-related scores correlating with GPX8 (***p < 0.001; **p < 0.01; *p < 0.05; ns. no significance)

Tumor microenvironment remodeling is considered to be one of the key factors affecting patient prognosis and anticancer therapy. Considering that TCGA-LUAD patients have abundant information about the properties of the defined tumor microenvironment, we analyzed the correlation between GPX8 and the tumor microenvironment in the TCGA cohort. We obtained tumor purity information of TCGA-LUAD patients from a previous study and performed correlation analysis with gene expression of GPX8. The results showed that GPX8 was negatively correlated with tumor purity (*R*=-0.295, *P* < 0.001) (Fig. 5A), suggesting that GPX8 was mainly expressed in non-tumor cells. We then used the ESTIMATE method to assess the patients’ immunity score, stromal score, and ESTIMATE score. Correlation analysis showed positive correlations between the immune score (*R* = 0.194, *P* < 0.001), stromal score (*R* = 0.415, *P* < 0.001) and ESTIMATE score (*R* = 0.328, *P* < 0.001) for GPX8, with the stromal score having the highest correlation with GPX8 (Fig. 5B).

Heatmaps showed the distribution of 29 known tumor microenvironmental signatures in relation to GPX8 expression. As expected, TME signatures such as cancer-associated fibroblast, matrix, matrix remodeling, and EMT signature rose with a gradual rise in GPX8 (Fig. 5C). CIBESORT analysis showed that in the high GPX8 group, regulatory T cells and activated dendritic cell infiltration were significantly increased (Fig. 5D). Among them, regulatory T cells are considered to be one of the major cell subsets promoting immune response suppression [43, 44].

Sankey diagram showed that the tumor microenvironment properties in the high expression GPX8 group were predominantly C2 immune subtype and F subtype predominant (Fig. 5E). Subsequent subgroup comparison plots similarly showed that GPX8 expression was most active in the F subtype as defined by Bagaev et al. and active in the C1 and C2 subtypes as defined by Thorsson et al. (Fig. 5F, G). In addition, in the TME features summarized by Zeng et al. we found that GPX8 was highly correlated with EMT1 and EMT2 (Fig. 5H). In conclusion, these results suggest that the tumor microenvironment with active GPX8 expression mainly exhibits immunosuppressive cell-enriched and fibroblast-enriched phenotypes as well as an active EMT program phenotype.

### GPX8 is associated with clinical treatment efficacy

**Fig. 6:**
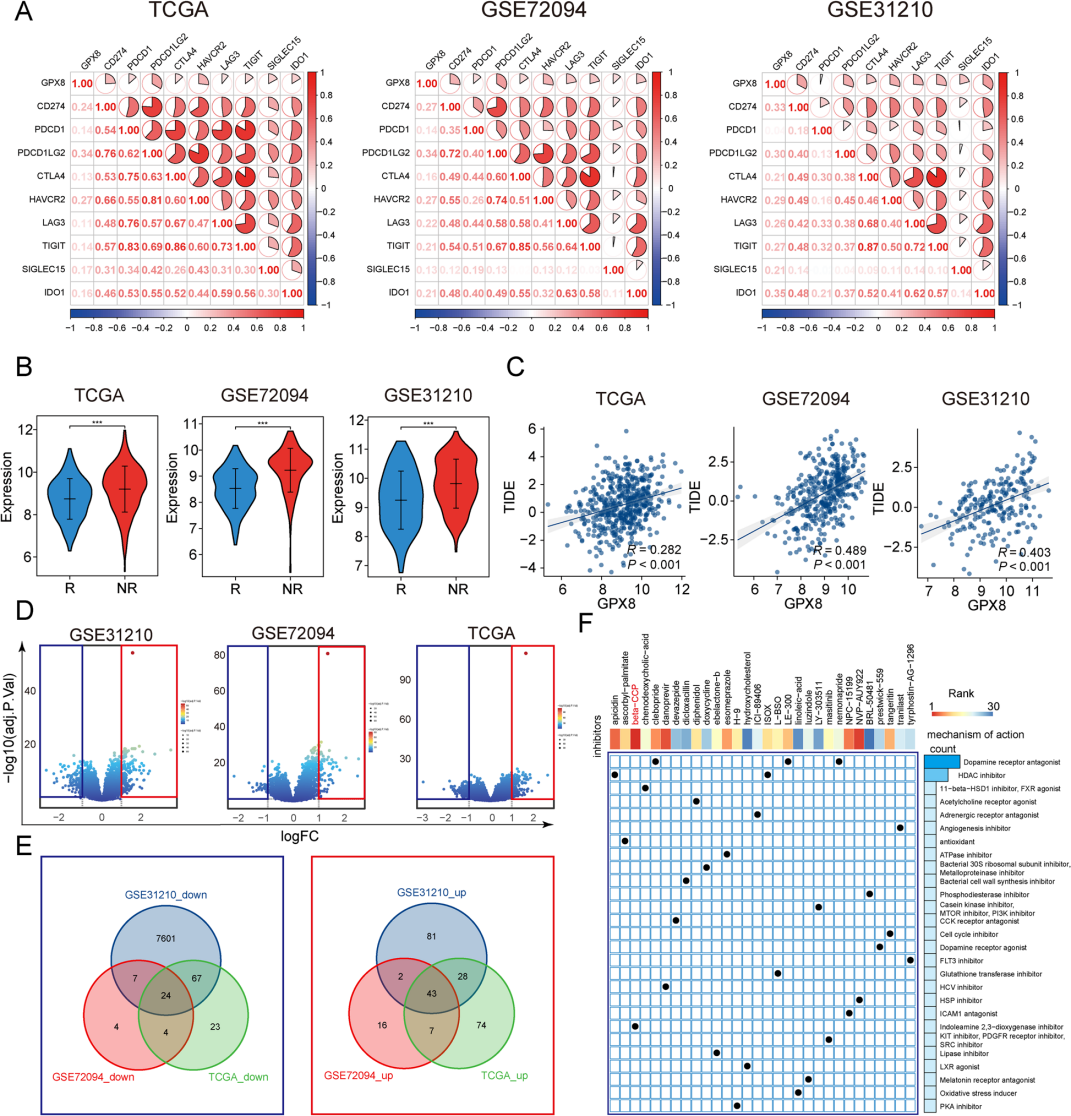
Clinical value of GPX8. **A** Heatmap of GPX8 correlation with immune checkpoints in 3 LUAD cohorts. **B** Distribution of GPX8 expression in immune-responsive and non-immune-responsive groups assessed based on the TIDE algorithm. **C** Correlation scatter plots of GPX8 with TIDE scores in the 3 LUAD cohorts. **D** Differential gene volcano plot based on high and low GPX8 expression groups. **E** Venn plots showing up- and down-regulated genes shared in the 3 cohorts (adj. *p* value < 0.05 and |logFC| > 1). **F** Small molecule drugs screened based on up- and down-regulated genes and the cMAP database, with the heatmap at the top ranking the small molecules

Immunotherapy, as a novel anticancer treatment, has achieved promising results in recent years. However, the limited number of beneficiary populations and the specificity of indications have been one of the limitations preventing its large-scale application [45]. Therefore, identifying effective immunotherapy biomarkers can help clarify the appropriate population for immunotherapy. In this study, we investigated the correlation between GPX8 and common immune checkpoints. The correlation heatmap demonstrated a positive correlation between GPX8 and immune checkpoints across all 3 LUAD cohorts (Fig. 6A). We further utilized the TIDE algorithm to evaluate the degree of immunotherapy response and immune escape in patients. Interestingly, non-responsive patients evaluated by the TIDE algorithm exhibited significantly higher levels of GPX8 expression (Fig. 6B). The correlation scatter plot confirmed a significant positive correlation between GPX8 expression and the TIDE score, reflecting the level of immune escape (Fig. 6C).

Next, to explore the potential clinical applications of GPX8, we first compared the differentially expressed genes between the GPX8 high expression group and the GPX8 low expression group. The volcano plot displayed the distribution of differentially expressed genes in the 3 LUAD cohorts (Fig. 6D). Then, we overlapped differential genes with adj. *p* value < 0.05 and |logFC| > 1, and identified 24 down-regulated genes and 43 up-regulated genes shared among the 3 LUAD cohorts (Fig. 6E). Next, we imported these genes into the cMAP database to search for drugs that affected the GPX8 expression profile and visualized the results (Fig. 6F). The most effective drug in perturbing GPX8 expression was the beta-CCP small molecule compound. This compound has the potential to be used as a therapeutic drug targeting GPX8 for the treatment of lung adenocarcinoma.

### Unique transcriptional features of GPX8^+^ CAFs

**Fig. 7:**
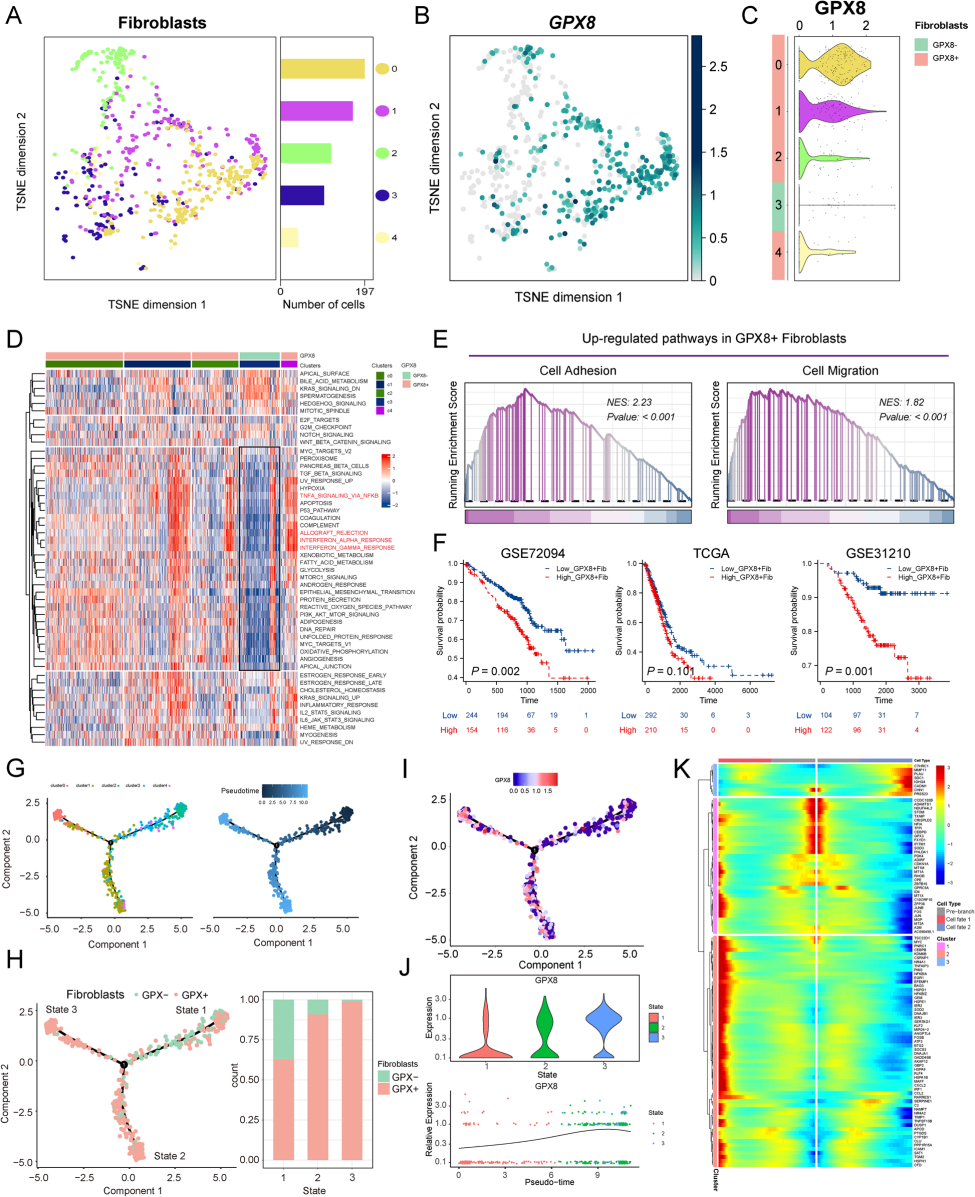
Functional analysis of GPX8^+^ CAFs. **A** t-SNE clustering plot of fibroblasts. Distribution of GPX8 expression in fibroblasts t-SNE plot (**B**) and violin plot (**C**). **D** Heatmap showing enrichment of hallmark gene sets in fibroblast subpopulations. **E** GSEA analysis for GPX8 ^+^ CAFs. **F** K-M curve analyzing the prognostic value of GPX8^+^ CAFs in LUAD. **G** The trajectory plot showed identified fibroblast clusters in pseudo timeline. **H** Trajectories showing the developmental trajectories of GPX8^−^ CAFs clusters and GPX8^+^ CAFs clusters. **I**-**J** Dynamic changes of GPX8 with cell developmental trajectories. **K** Heatmap of gene expression changes along cellular trajectories

Next, we extracted fibroblast subgroups from the GSE123902 dataset and applied clustering analysis. The results revealed the division of fibroblasts into five clusters (Fig. 7A), with cluster 3 showing weaker GPX8 expression compared to other cell subpopulations (Fig. 7B, C). Consequently, we categorized clusters 0, 1, 2, and 4 as GPX8^+^ fibroblasts, while cluster 3 was classified as the GPX8^−^ fibroblast subpopulation. The expression distribution of hallmark gene sets was then analyzed in the GPX8^+^ and GPX8^−^ fibroblast. The findings demonstrated significant enrichment of inflammation-related pathways, including INTERFERON ALPHA RESPONSE, INTERFERON GAMMA RESPONSE, ALLOGRAFT_REJECTION, and TNFA_SIGNALING_VIA_NFκB, in GPX8^+^ CAFs compared to GPX8^−^ CAFs (Fig. 7D). Furthermore, GSEA analysis indicated a substantial enrichment of differential genes in the GO_BP pathway of cell adhesion and cell migration (Fig. 7E). Similar results were observed in another LUAD single-cell dataset (Supplementary Fig. 6A-E).

To assess the relationship between GPX8^+^ fibroblasts and prognosis, we selected GPX8^+^ upregulate genes with avg_logfoldchange > 1, and quantified them in 3 bulk-RNA cohorts using the ssGSEA method, and examined their association with prognosis. The results indicated that high expression of GPX8^+^ CAFs correlated with poor prognosis in all 3 independent bulk-RNA cohorts (Fig. 7F). Considering the functional heterogeneity and poor prognosis of GPX8^+^ CAFs, we performed pseudotime analysis to investigate the underlying mechanism of functional changes. The temporal analysis revealed that cluster 3 projected to the root (state1), while cluster 0 (state3) and cluster 1 (state2) mainly projected to the two branches, respectively (Fig. 7G). Notably, GPX8^−^ CAF was primarily in state1 and gradually transitioned to GPX8^+^ CAF (state2 and state3) (Fig. 7H). In this process, GPX8 gene expression also changed with CAF state (Fig. 7I-J). Subsequently, we conducted BEAM analysis to identify branch-dependent genes. The results showed that genes such as MMP11 and SDC1 were positively correlated with the developmental direction of GPX8^+^ CAFs (Fig. 7K).

## Discussion

CAFs, one of the key components of the tumor microenvironment, are thought to be associated with a higher risk of immune evasion and a poorer prognosis. To gain insight into the role of CAFs in the progression mechanism of LUAD and to identify potential therapeutic targets, we combined single-cell sequencing data analysis with bulk-RNA sequencing data analysis to reveal the potential value of GPX8^+^ CAFs as biomarkers for prognosis and indicators of immunotherapy in LUAD.

Imbalanced shaping of the tumor microenvironment (TME) is a major factor contributing to the poor prognosis of cancer patients. The TME is composed of a variety of stromal cells, with cancer-associated fibroblasts (CAFs) being the most abundant [10]. CAFs can influence tumor progression by regulating changes in the composition of the TME through cell-to-cell contacts, secretion of regulatory molecules, and extracellular vesicles [46]. In this study, single-cell sequencing data revealed that CAFs were more enriched in tumor tissues compared to normal tissues, and survival analyses showed a significant correlation between CAFs and poor patient survival in bulk-RNA data. Similarly, a pan-cancer study by Luo et al. found that in terms of interactions between TME components, fibroblasts dominated interactions with other TME components, with a progressive increase in the order of normal, neighboring, and tumor samples in the dimension of single-cell resolution [47]. These results suggest a possible increased recruitment and generalized activation of fibroblasts in the TME, which may contribute to the poor prognosis of cancer.

Subsequently, we identified GPX8 as a CAFs-associated biomarker based on comprehensive bioinformatics analyses. GPX8 is a localized ER transmembrane protein that regulates ROS production and clearance in organisms [19]. Recent studies have shown that GPX8 is associated with poor prognosis in various cancers, including gastric cancer, lung cancer, and glioma [23, 25, 48]. In clear cell renal cell carcinoma, GPX8 regulates NNMT expression through the IL6-STAT3 signaling axis and inhibits ccRCC cell survival by blocking this axis through activation of AMPK [23]. Similarly, in esophageal squamous carcinoma cells, GPX8 was found to induce the onset of autophagy and apoptosis by regulating the IRE1/JNK pathway in tumor cells [49]. These findings collectively suggest a connection between GPX8 expression in tumor tissues and cancer progression.

Notably, in a recent study on lung cancer, researchers discovered that GPX8 expression on cancer-associated fibroblasts can promote the metastasis of lung cancer cells [50]. Our findings, corroborated in multiple cohorts, indicate that in LUAD, GPX8 is primarily localized on CAFs. Consistent with our expectations, GPX8 was significantly associated with reduced survival time and exhibited a strong predictive effect on prognosis in multiple independent LUAD cohorts. Even after adjusting for confounding factors, GPX8 remained an independent prognostic predictor in lung adenocarcinoma patients. These findings suggest that GPX8 has the potential to serve as a valuable biomarker for LUAD. Furthermore, we observed that GPXs family members, including GPX8, commonly exhibit copy number variation (CNV) changes across various cancers. This suggests that the abnormal function of these genes in the carcinogenesis process may be linked to DNA damage. Interestingly, Chu et al. found in a mouse model that the disruption of GPX1 and GPX2 genes increased the mice’s susceptibility to certain cancers [51]. Collectively, these results indicate that genetic variants within the GPXs family may contribute to tumor formation and partly account for the association between GPX8 and aggressive tumor characteristics.

Regarding tumor microenvironment expression patterns, we observed a significant correlation between GPX8 and the infiltration of non-tumor cellular components. This suggests a higher occurrence of cell-to-cell signaling crosstalk in TMEs with high GPX8 expression. Interestingly, apart from the enrichment of cancer-associated fibroblasts in GPX8 high expression TMEs, we also found the active expression of the epithelial-mesenchymal transition (EMT) program. Coincidentally, a study by Khatib has reported that GPX8 expression is crucial for maintaining the EMT phenotype in breast cancer cells [23]. Furthermore, numerous studies have highlighted the signaling crosstalk between EMT progression and immune escape. Wang et al. conducted transcriptomic studies at the pan-cancer level, revealing the existence of signaling crosstalk between EMT progression and immune escape [52]. Alsuliman et al. demonstrated an association between the progression of the EMT program and PD-L1-mediated immune escape in breast cancer cells [53]. This partly explains the intrinsic link between GPX8 and the occurrence of immune escape. In our study, we found that the proportion of Treg cell infiltration was upregulated in TME with high GPX8 expression. Treg cells, as immunosressive T cells, are primarily responsible for downregulating immune and inflammatory responses and maintaining organismal stability. In tumors, the recruitment of Treg cells is closely associated with tumor immune escape [43, 44]. Immune checkpoints, as an important part of promoting the occurrence of immune escape, have entered the field of clinical treatment with a large number of immune checkpoint blockers, and combination therapy with immune checkpoint blockade has become one of the main anticancer therapeutic tools in the clinic [54, 55]. Our correlation analysis revealed that GPX8 positively correlates with immune checkpoints such as PD-L1 and CTLA-4 in several LUAD cohorts. Furthermore, in the context of immunotherapy, patients with high GPX8 expression demonstrated a significantly lower response rate to immunotherapy compared to those with low GPX8 expression. Collectively, these findings indicate that GPX8 is associated with the formation of an immunosuppressive microenvironment and could represent a potential therapeutic target for immunotherapy.

To expand the potential clinical applications of GPX8, we conducted a screening for beta-CCP using the cMAP database. Previous research has demonstrated that beta-CCP can antagonize diazepam-induced passive avoidance disorder [56]. Our study revealed that beta-CCP exhibited the most significant inhibitory effect on GPX8-related expression profiles. These findings suggest that beta-CCP may be a promising therapeutic agent for the treatment of LUAD.

Finally, we delved into the potential function of the GPX8^+^ CAFs subpopulation in LUAD. We observed that the GPX8^+^ CAFs cell subpopulation displayed a significantly more active inflammation-related pathway compared to the GPX8^−^ CAFs cell subpopulation. Additionally, the presence of the GPX8^+^ CAFs subpopulation was strongly associated with poor prognosis. These findings suggest that the GPX8^+^ CAFs subpopulation may exert its influence on cancer progression by participating in immune regulation within the tumor microenvironment (TME). It is worth mentioning that Öhlun et al. categorized CAFs in pancreatic cancer into myofibroblast and inflammatory fibroblast based on phenotypic and molecular heterogeneity of CAFs [57]. Among them, the phenotype of inflammatory CAFs is mainly characterized by fibroblasts exhibiting active immunoregulatory pathways. In bladder uroepithelial carcinoma, enrichment of inflammatory CAFs was significantly associated with poor patient prognosis [58]. Cell trajectories showed an upregulation of GPX8 expression in response to changes in fibroblast activity. We hypothesized that the upregulation of GPX8 may drive the transition of CAFs to the inflammatory fibroblast phenotype. However, the underlying mechanisms still need to be further explored. Overall, GPX8^+^ CAFs may have an important role in remodeling TME, and targeting GPX8^+^ CAFs may serve as a promising novel therapeutic strategy for LUAD.

The study still has several limitations, the data used in the study are mainly from public datasets, and further experiments are still needed for exploration and validation. The lack of large-scale clinical specimens for the validation of the results is also one of the limitations of this paper. In addition, we will also try to construct an internal LUAD cohort in the future to validate and explore the results of the current analysis in depth.

## Conclusion

In this study, we identified GPX8 as a key prognostic gene expressed in CAFs through a series of bioinformatics analysis. And the high expression of GPX8 was associated with poor patient survival and the formation of an immunosuppressive microenvironment. Consequently, GPX8^+^ CAFs holds potential as a therapeutic target for LUAD.

### Supplementary Information

**Supplementary material 1.**

## Data Availability

The datasets analyzed during the current study are available in the UCSC website (https://xenabrowser.net/datapages/, TCGA-LUAD) and GEO repository, (https://www.ncbi.nlm.nih.gov/geo, accession number GSE31210, GSE72094, GSE30219, GSE50081, GSE19188, GSE123902 and GSE153935).
