# Supplementary material for: GPX8+ cancer-associated fibroblast, as a cancer-promoting factor in lung adenocarcinoma, is related to the immunosuppressive microenvironment
Source: BMC Med Genomics. 2024 Mar 21;17:77. doi: 10.1186/s12920-024-01832-8 (PMC10958965; doi:10.1186/s12920-024-01832-8)
Supplement: Supplementary file 1 — Supplementary material 1. [file 12920_2024_1832_MOESM1_ESM.docx]

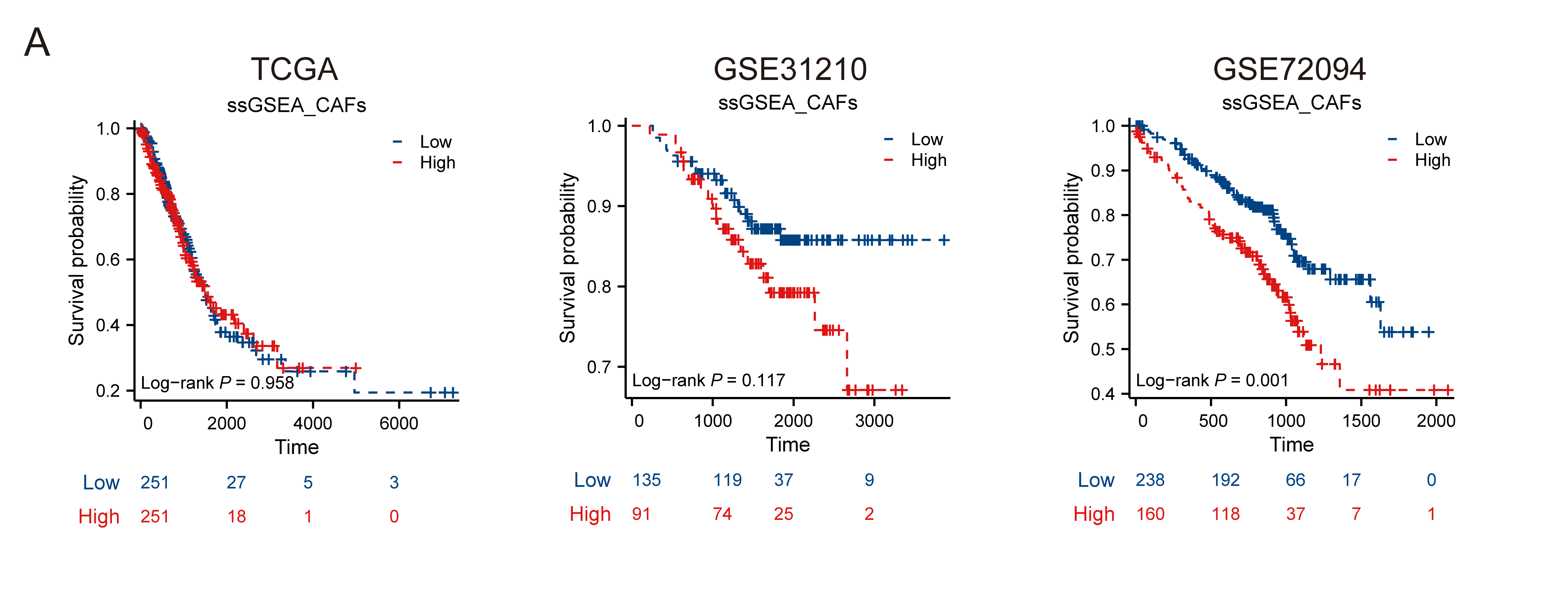
**Supplementary Fig.1** **Prognostic role of fibroblasts.** (A) Survival analysis of ssGSEA_CAFs in TCGA, GSE31210 and GSE72094 datasets


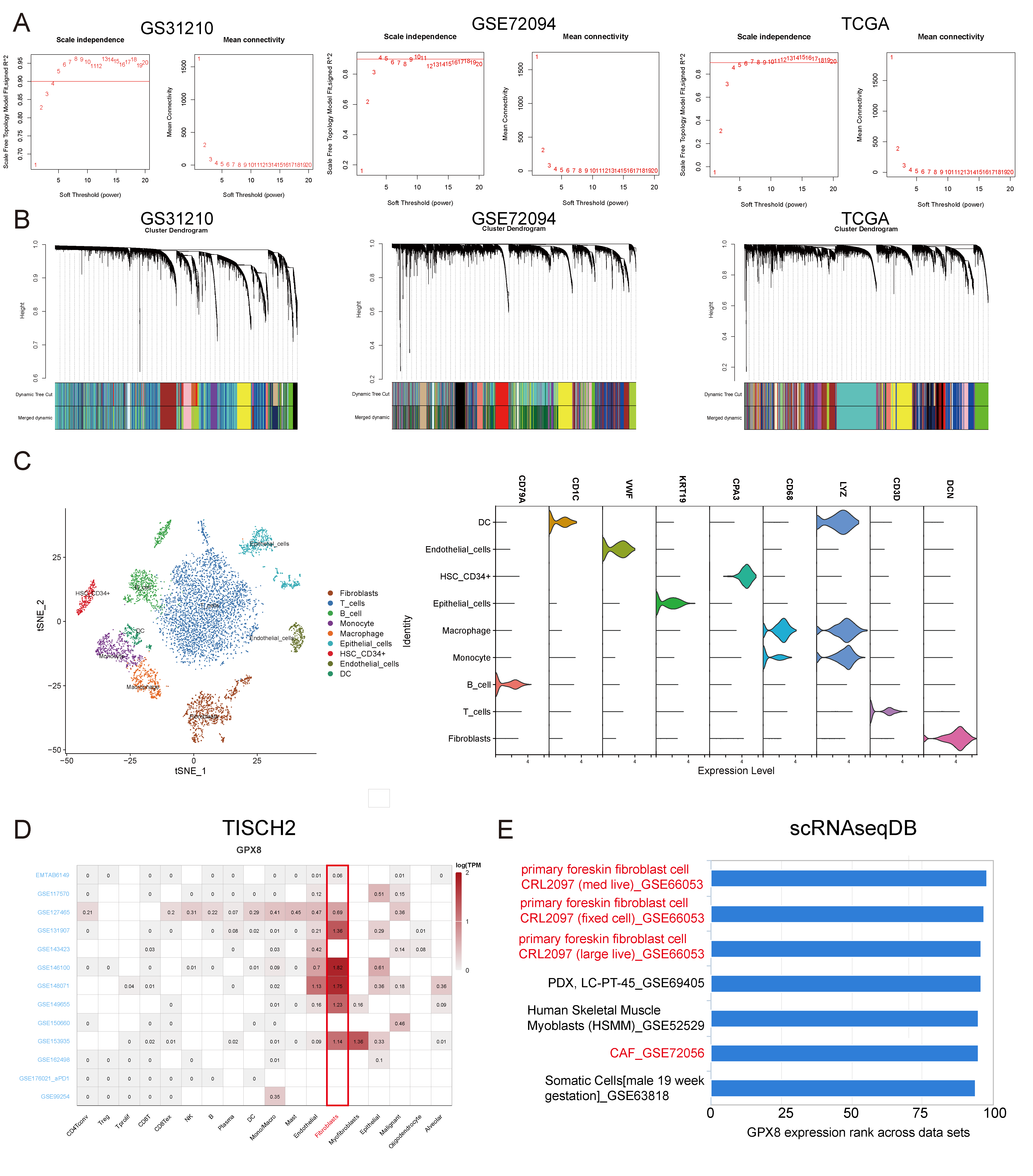


**Supplementary Fig.2 Screening for key genes in CAFs.** (A) Analyze the scale-free fit index and the average connectivity of the 1-20 soft threshold power (β) in 3 LUAD cohorts. (B) Hierarchical clustering tree of genes in various modules. (C) Cell clustering map and violin map of signature gene expression for the GSE153935 dataset. (D) TISCH2 database showing the cell clustering and expression levels of GPX8 in various cells in LUAD datasets. (E) scRNASeqDB database showing the expression levels of GPX8 in various cells.


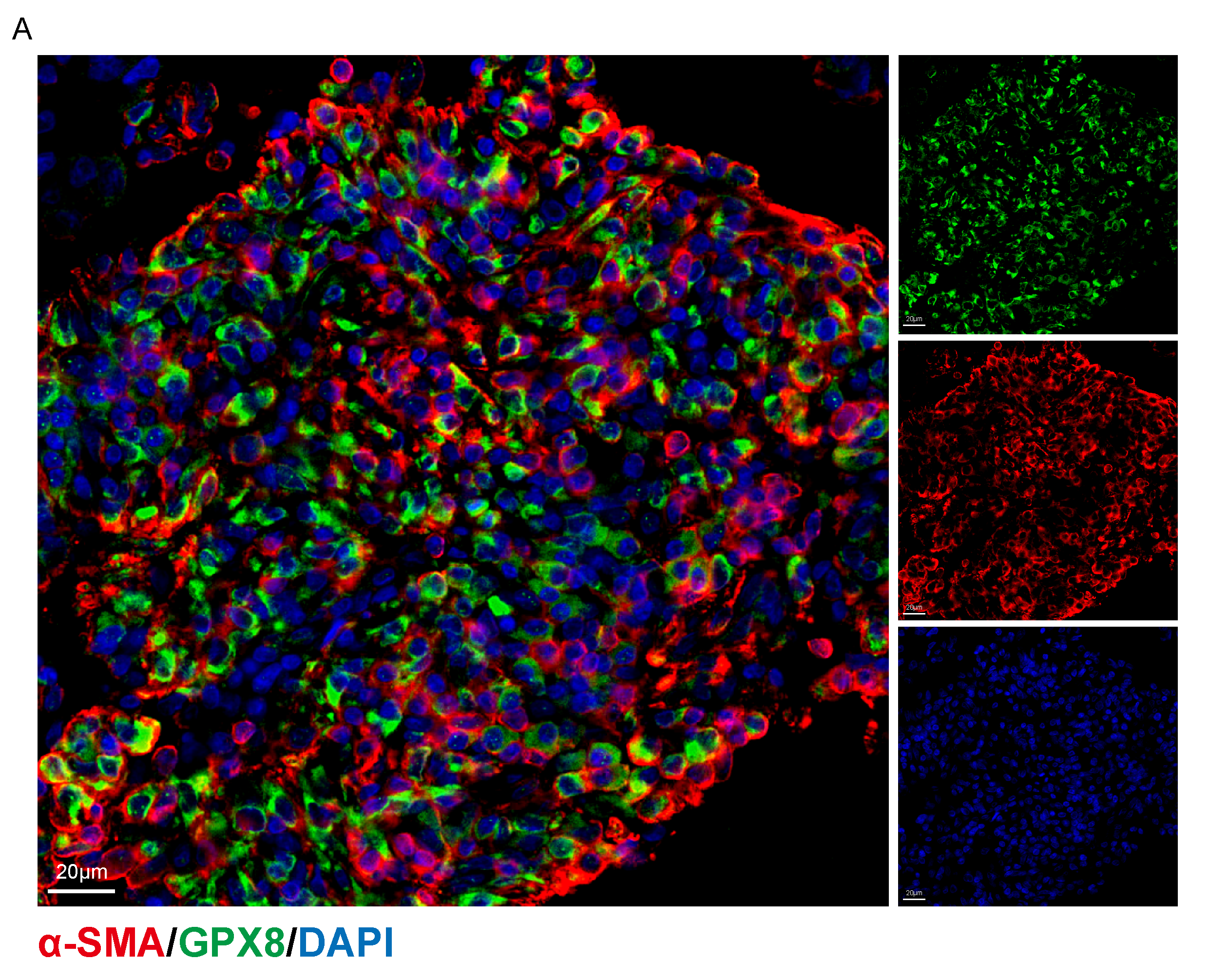
**Supplementary Fig.3 Validation of GPX8 expression.** (A) Immunofluorescence staining of LUAD tissues.


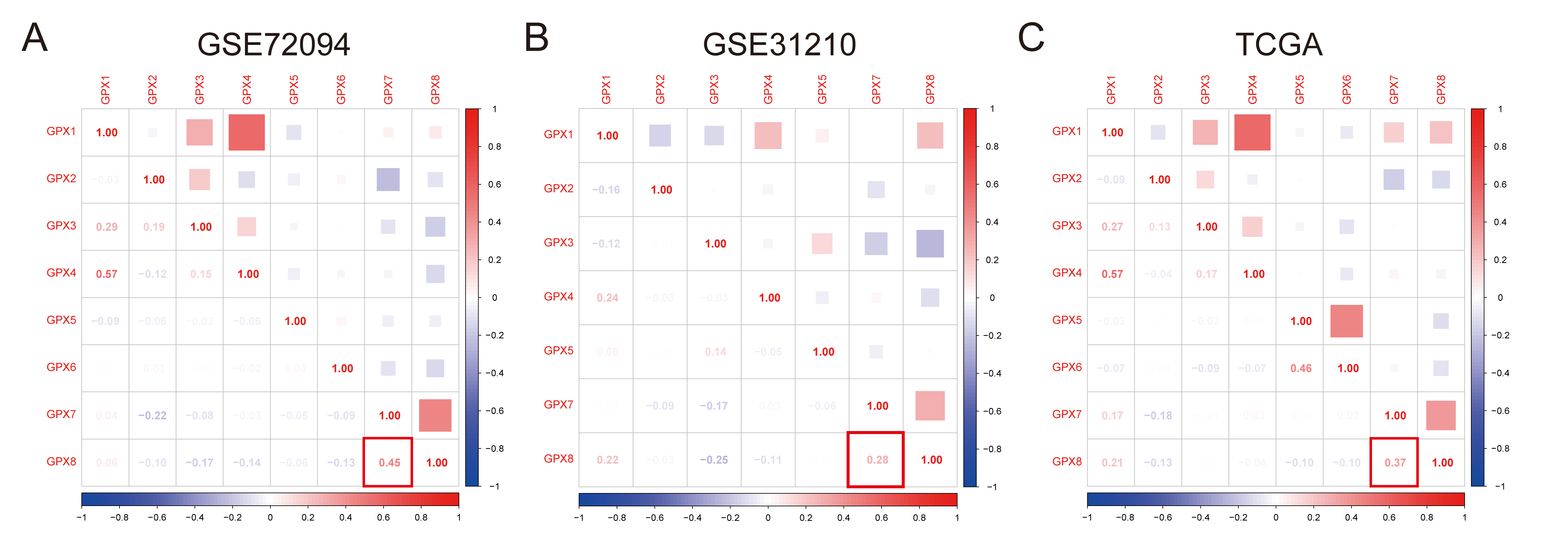


**Supplementary Fig.4 Correlation analysis of the GPXs family.** Heatmap of GPX family correlations in GSE72094 (A), GSE31210 (B), and TCGA (C) cohorts.


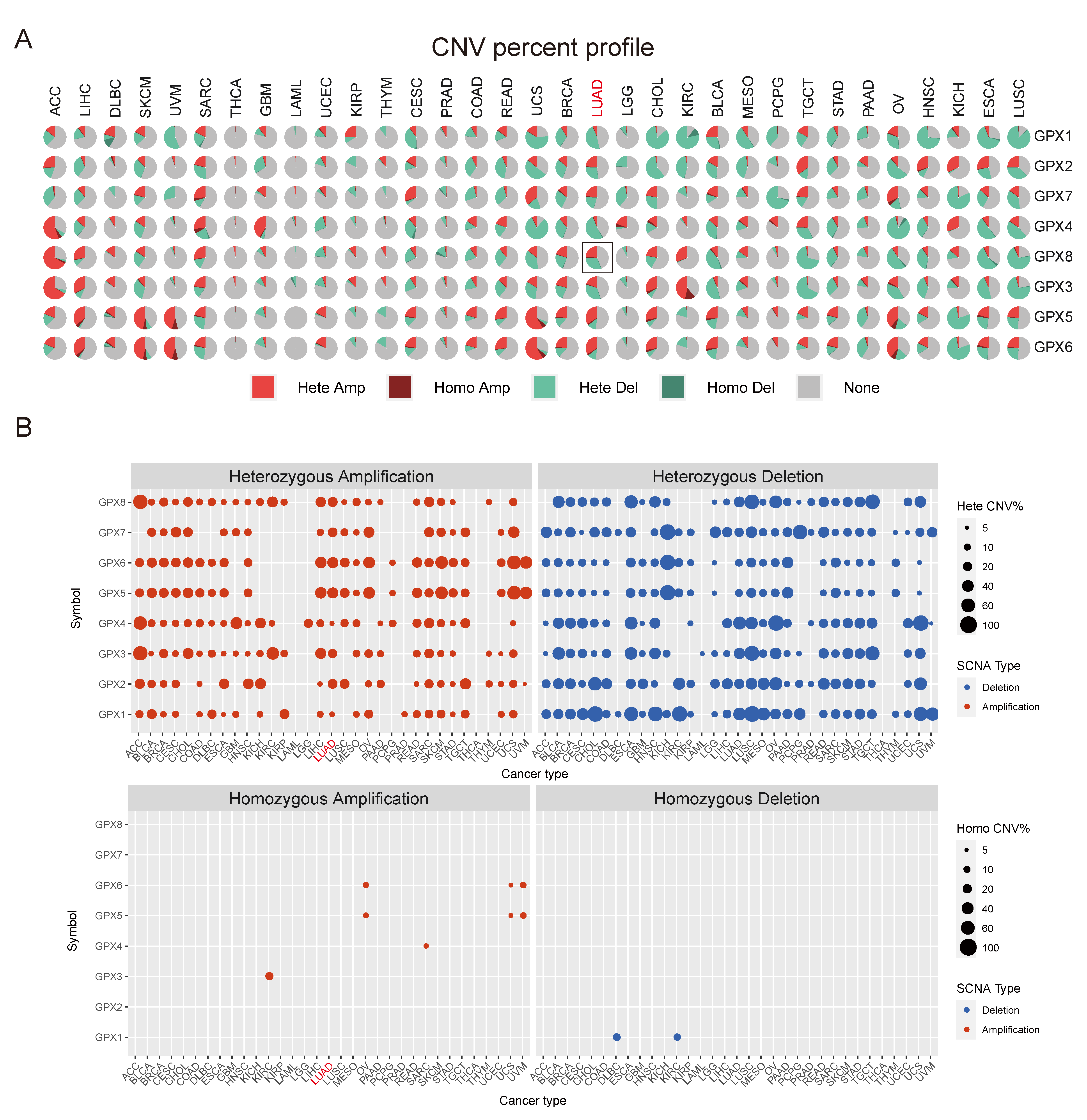


**Supplementary Fig.5 CNV of the GPXs family in pan-cancers.** (A) The CNV pie distribution indicates the constitution of Heterozygous/Homozygous CNV of the GPXs family in pan-cancer. (B) The Heterozygous/Homozygous CNV of the GPXs family in pan-cancer.


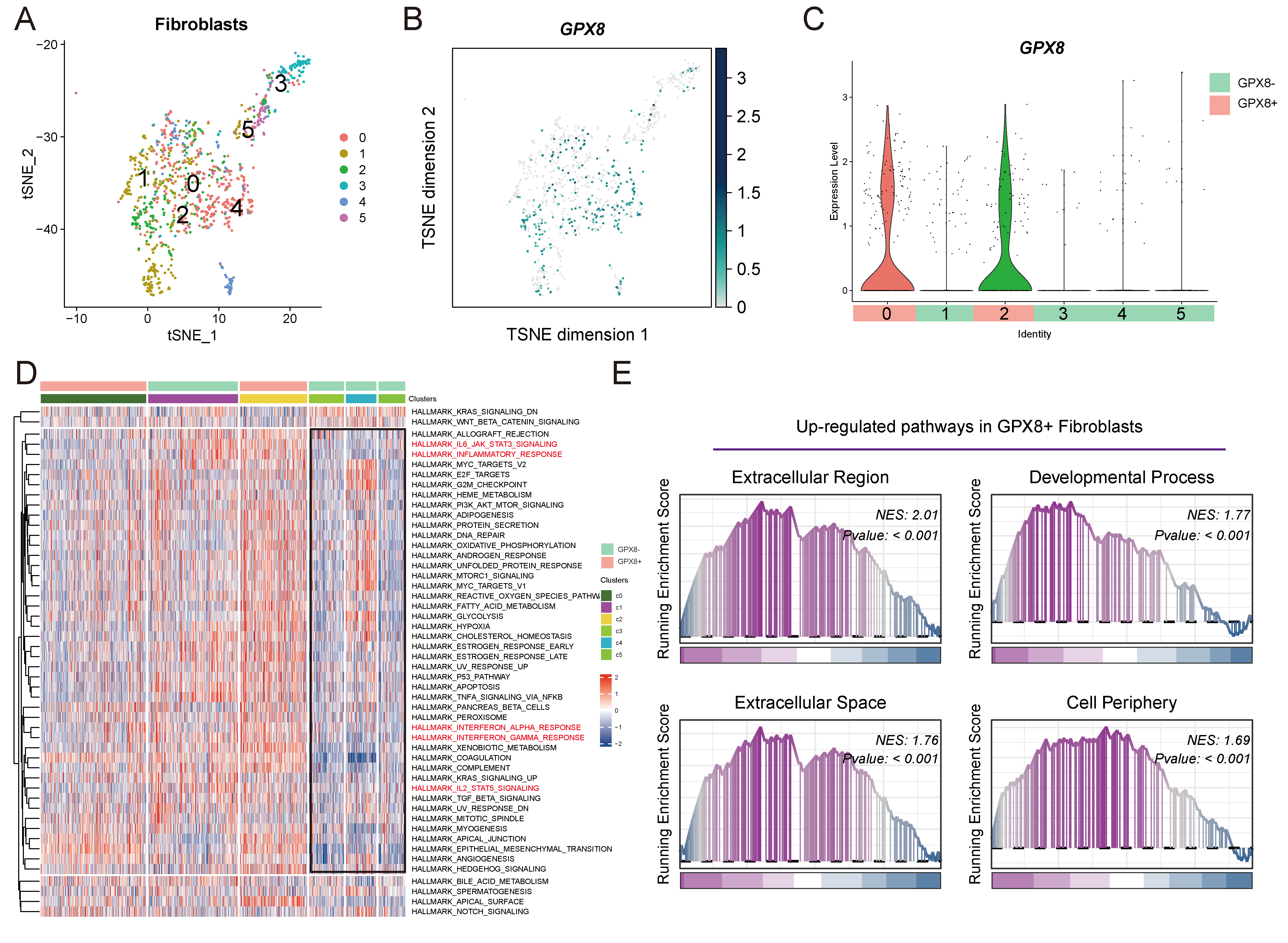


**Supplementary Fig.6 Molecular characterization of GPX8^+^ CAF based on the GSE153935 dataset.** (A) t-SNE clustering plot of fibroblasts. Distribution of GPX8 expression in fibroblasts t-SNE plot (B) and violin plot (C). (D) Heatmap of the enrichment of hallmark gene sets in fibroblast subpopulations. (E) Biological pathways involved in GPX^+^ CAFs.
